# Evapotranspiration Measurement and Crop Coefficient Estimation over a Spring Wheat Farmland Ecosystem in the Loess Plateau

**DOI:** 10.1371/journal.pone.0100031

**Published:** 2014-06-18

**Authors:** Fulin Yang, Qiang Zhang, Runyuan Wang, Jing Zhou

**Affiliations:** 1 Key Laboratory of Arid Climatic Change and Reducing Disaster of Gansu Province, Key Open Laboratory of Arid Climatic Change and Disaster Reduction of China Meteorological Administration (CMA), Institute of Arid Meteorology, CMA, Lanzhou, China; 2 State Key Laboratory of Grassland Agro-ecosystems, College of Pastoral Agriculture Science and Technology, Lanzhou University, Lanzhou, China; Agroecological Institute, China

## Abstract

Evapotranspiration (ET) is an important component of the surface energy balance and hydrological cycle. In this study, the eddy covariance technique was used to measure ET of the semi-arid farmland ecosystem in the Loess Plateau during 2010 growing season (April to September). The characteristics and environmental regulations of ET and crop coefficient (Kc) were investigated. The results showed that the diurnal variation of latent heat flux (LE) was similar to single-peak shape for each month, with the largest peak value of LE occurring in August (151.4 W m^−2^). The daily ET rate of the semi-arid farmland in the Loess Plateau also showed clear seasonal variation, with the maximum daily ET rate of 4.69 mm day^−1^. Cumulative ET during 2010 growing season was 252.4 mm, and lower than precipitation. Radiation was the main driver of farmland ET in the Loess Plateau, which explained 88% of the variances in daily ET (p<0.001). The farmland Kc values showed the obvious seasonal fluctuation, with the average of 0.46. The correlation analysis between daily Kc and its major environmental factors indicated that wind speed (Ws), relative humidity (RH), soil water content (SWC), and atmospheric vapor pressure deficit (VPD) were the major environmental regulations of daily Kc. The regression analysis results showed that Kc exponentially decreased with Ws increase, an exponentially increased with RH, SWC increase, and a linearly decreased with VPD increase. An experiential Kc model for the semi-arid farmland in the Loess Plateau, driven by Ws, RH, SWC and VPD, was developed, showing a good consistency between the simulated and the measured Kc values.

## Introduction

Water cycle is the key process in the multi-layers interaction of earth system. Land surface evapotranspiration (ET), as the important segment of water cycle, is the main way for water consumption of earth system, playing an important role in regional and global climate [1], [2]. Farmland ET refers to the overall water flux sent to the air by vegetation and earth surface, which is the important component of water balance. About 60% rainfall and 99% water in farmland system are consumed by ET around and the world [3]. Furthermore, as a component of energy balance, ET is also the important consumption of surface available energy [1]. Farmland irrigation and ET can occupy 60∼80% of the net radiation during the growing season [4].

Recently, a series studies were carried out to investigate the characteristics of farmland ET, suing process-based models, remote sensing [5], calculation, and direct observation [6],[7]. By applying eddy covariance technology, Zhang *et al*. [8] found that most of net radiation was consumed by crop latent heat during the growing period, and higher ratio between ET and net radiation for summer corn (*Zea mays* L.) than that of winter wheat (*Triticum aestivum* L.) in Licheng area. Yang *et al*. [9] and Li *et al*. [10] investigated the effects of net radiation (Rn), soil water content (SWC), leaf area index (LAI) and rainfall seasonal distributions to the daily ET changes in corn ecosystem. On the other hand, the empirical models, such as Priestley–Taylor [11] and crop coefficient (Kc) methods [12], were mostly used to estimate the crop actual ET.

Loess Plateau is the unique land type and ecological environmental region in China, has a total area of 0.63 million km^2^ with nearly 70% covered with thick loess. Most regions of the Loess Plateau receive less annual precipitation with large precipitation variation and suffer serious spring drought. Furthermore, the Loess Plateau is the climate demarcation area in China and the main distribution area of ecotone between agriculture and animal husbandry, an important and unique region whose water cycle will exert significant impact on the atmospheric circulation and agriculture development in East Asia. However, water resources shortage becomes the current bottleneck that restricts the agricultural sustainable development. Studies indicated that the Loess Plateau is the sensitive area to climate change as climate warming will aggravate the soil drought by accelerating the water exchange process [13]. ET, as the critical process of water circulation, had become an important scientific issue in agricultural sustainable development and impacts of climate change. Knowledge of the farmland ET process in the Loess Plateau is an important research content in water and energy balance of the terrestrial ecosystem, and promoting the reasonable utilization of limited water resources in the Loess Plateau.

Several measurement researches on the farmland ET in the Loess Plateau by lysimeter [14] or eddy correlation system [15] are become available. Moreover, Li [16], [17] compared the adaptability of different ETo estimation methods in the Loess Plateau area and analyzed the time-space variation characteristics of crop reference evapotranspiration (ETo) in the Loess Plateau based on Penman-Monteith model. Zhang *et al*. [18] compared the differences of observing methods for land surface ET in the Loess Plateau, showing that the actual ET estimated based on Penman-Monteith model and crop coefficient is significantly lower than that observed through eddy covariance method and lysimeter.

The spring wheat is the main grain crop in the semi-arid rain-fed region of Loess Plateau. Precipitation and ET are critical for spring wheat, which is the main water-demande crop of region, because the irrigation and infiltration were unavailable completely. An accurate estimate of crop ET is required for appropriate water management in order to increase water uses efficiency in the water-limited region [12]. Crop coefficient method was recommended by Food and Agriculture Organization of the United Nations to estimate the ET amount in the different terrestrial ecosystems, which is widely applied in various ecological systems, such as crops, grassland ecosystem [19], [20]. In terms of Kc method, it is the key to obtain specific reliable Kc value to apply Kc method for ET estimation accurately. Some studies revealed that Kc not only has significant differences in different types of ecological system, but also is influenced by various environment factors [20], [21]. However, most current researches regard Kc as a constant or suppose the Kc during the specific crop growing period as a constant value, ignoring its daily variation [18]. The approximate treatment on Kc values will influence the accuracy of ET estimation. Therefore, it is of much significance for ET dynamic simulation to investigate Kc characteristics of different underlying surfaces.

In this study, we carried out an successive ET observation in semi-arid farmland ecosystem in the Loess Plateau during 2010 growing season (from 1^st^ April to 30^th^ September) by eddy covariance system, analyzed the dynamics of farmland ET and Kc, and then developed an empirical Kc model.

## Materials and Methods

### 1.1 Ethics Statement

No specific permits were required for the described field studies. The location is not privately-owned or protected in any way during the study period, and the field studies did not involve endangered or protected species in these areas.

### 1.2 Study Site

The study site carried out at Dingxi Arid Meteorology and Ecological Environment Experimental Station (35°33′ N,104°35′ E, 1896.7 m a.s.l), where located in the west of Dingxi city in Gansu Province, China. This area belongs to semi-arid temperate continent climate with an annual average temperature of 7.1°C. The lowest average monthly temperature is 7.0°C (January) and the highest average monthly temperature is 19.0°C (July). It has an average annual precipitation of 382.5 mm (1979 to 2008, from the Dingxi meteorological station), with most of the precipitation (80%) falling between May and September. It has flat and homogenous underlying surface, and the soil type is loess-like loam with an average bulk density of 1.38 g cm^−3^. The dominate crops was the spring wheat (*Triticum aestivum* L. cv. ‘Dingxi 24’).

### 1.3 Water and Heat Fluxes Measurements

Although there are several methods in ET measurements currently, the eddy covariance technology has become one of prior measure approach in determining the water exchange between atmosphere and land ecological system boundary layer due to its advantages of less theoretical assumptions and high measurement accuracy, and the measure data are often used to examine the simulation accuracy of model [7], [22]. The farmland ET in semi-arid area of the Loess Plateau was measured by eddy covariance in this study. The eddy covariance system, composed of three-dimensional sonic anemometer (CSAT-3, Campbell Scientific, USA) and an open path infrared gas analyzer (IRGA, Li-7500, LI-COR, USA), is mainly applied for measuring the exchange of latent heat flux and sensible heat flux between surface and atmosphere. The sampling frequency was set as 10 Hz and the real-time observed data was recorded by the data logger (CR5000, Campbell Scientific, USA).

The positive values of latent heat flux and sensible heat flux represents that energy transferred from land surface to atmosphere, while the negative values mean opposite. Before data calculation, the latent heat flux and sensible heat flux were treated with spike detection and removal and coordinate rotation were performed. In addition, sonic temperature fluctuations were taken into account to correct the fluxes of sensible heat; the Webb-Pearman-Leuning correction was used to adjust density changes resulting from fluctuations in latent heat [23]. Furthermore, all anomalous values of latent heat flux and sensible heat flux were deleted through following the criteria [20]: (1) the incomplete measuring data caused by power failure, instrument calibration, and so on; (2) precipitation events; (3) anomalous data detected by Papale *et al*. [24]. About 15.9% of EC flux data within the observation period in 2010 were deleted. Falge *et al*. [25] method was applied to fill the data gaps.

### 1.4 Environmental Factors Measurements

The experimental site was equipped with an automatic weather system (Campbell Scientific, USA) to measure environmental factors on the semi-arid farmland ecosystem in the Loess Plateau, including precipitation (PPT) (52203, RM Young, USA), net radiation (Rn) (CNR-1, Kipp & Zonen, Netherlands), air temperature (Ta), air relative humidity (RH) (HMP45C, Vaisala, Finland), wind speed (Ws) at height of 2 m, and soil water content (SWC) at 10 cm depth (CS616, Campbell Scientific, USA). The sampling frequency of both common meteorological and soil environmental factors was set as 0.1 Hz, and the data acquisition unit (CR1000, Campbell Scientific, USA) was applied for data storage. The half-hourly soil heat fluxes were calculated through Ts and SWC data, which were expressed in Yang *et al*. [26] in detail.

### 1.5 Reference Evapotranspiration and Crop Coefficient

The daily ETo of the semi-arid farmland in the Loess Plateau was calculated by Penman-Monteith model, as following [19]:
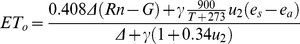
(1)where ETo is the reference ET, Rn is net radiation; G is soil heat flux; *γ* is the psychrometric constant (kPa°C^−1^); Ta is air temperature (°C); u is wind speed; e_s_ is saturation vapor pressure; e_a_ is actual vapor pressure; e_s_-e_a_ represents the differential saturation vapor pressure; and Δ is the slope of saturation vapor pressure curve (kPa°C^−1^). Crop actual water consumption (ET) can be calculated from referential evapotranspiration (ETo) and Kc [27]. Numerically, crop coefficient is the ratio of ET to ETo [19]:
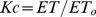
(2)ET measured by eddy covariance directly was regarded as ET (mm), while the calculation result obtained through Penman-Monteith model was regarded as ETo (mm).

### 1.6 Statistical Parameters

A quantitative evaluation on the fitting effect of ET model was conducted by using root-mean-square error, index of agreement, coefficient of determination and regression coefficient through the origin. The more the root-mean-square error close to 0, and the more the index of agreement, coefficient of determination and regression coefficient through the origin close to 1, that indicates the better the fitting effect of the model [20].

## Results and Discussion

### 2.1 Characteristics of Environmental Factors

**Figure 1:**
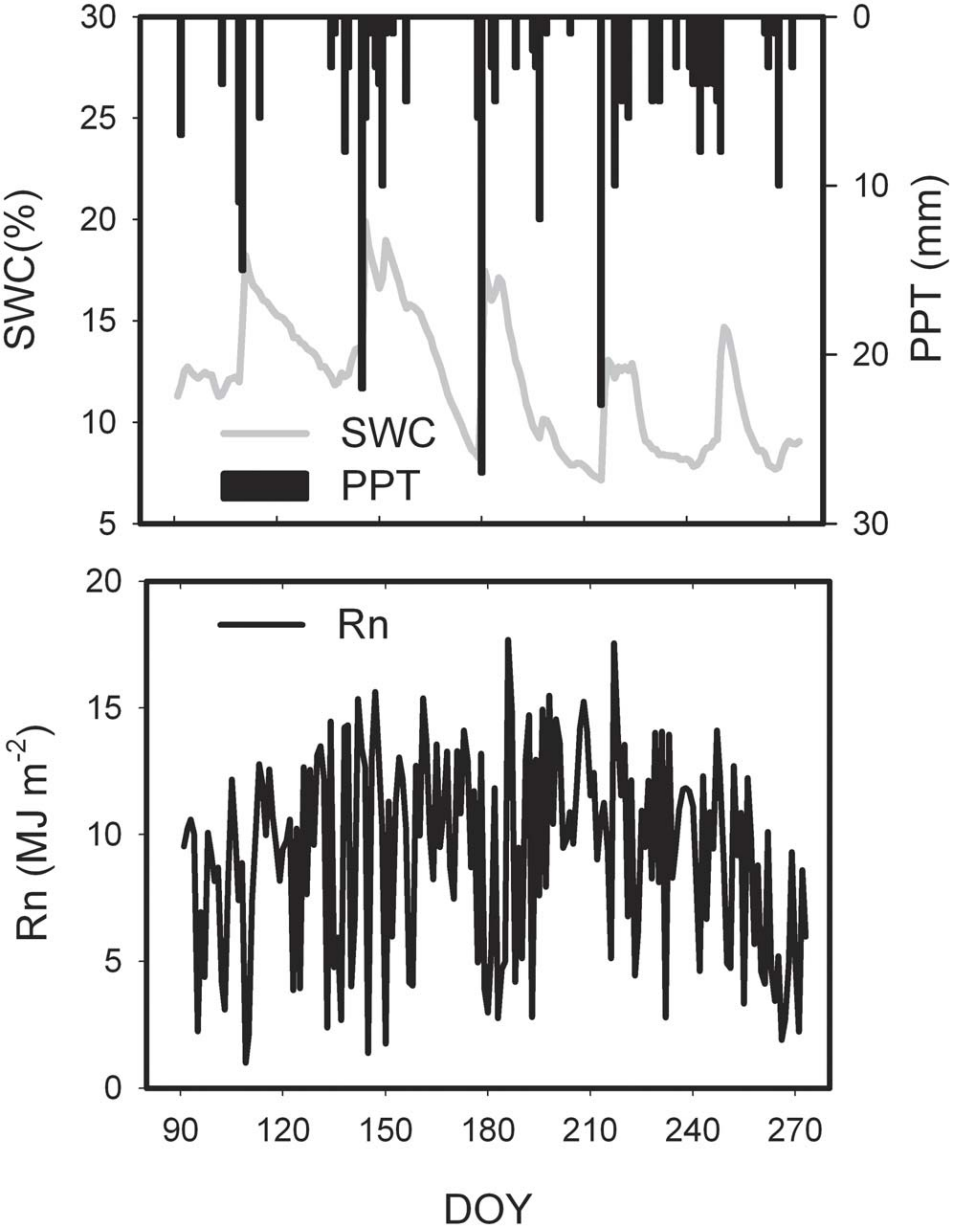
Seasonal variation of environmental factors over the semi-arid farmland ecosystem in the Loess Plateau.

The seasonal variation of PPT, SWC, and Rn from April to September are presented in Figure 1. There was totally 285.9 mm precipitation in the whole observation period, with the maximum daily precipitation of 26.9 mm (DOY 180). There had a little rainfall during early to middle of May as well as middle and late June, appearing obvious periodic drought. SWC was very sensitive to rainfall as a daily precipitation, especially the over 10 mm rainfall events. The seasonal variation of SWC showed significant fluctuation during the observation period, and SWC ranged from 7.2% to 19.9%, with a mean value of 12.0%. An obvious drought was appeared in the farmland ecosystem during the middle and late June. There was no rainfall record for 18 days successively, from 10^th^ to 27^th^ June, and the SWC on 27^th^ June, with only valued 8.5%. This serious drought decreased the RH significantly, but greatly increased atmospheric vapor pressure deficit (VPD) during this period. RH and VPD produced approximate opposite variation trend within the observation period. Furthermore, Rn and Ta showed obvious seasonal variations. Although the Rn in July was relative higher, it produced relative greater daily fluctuation during the whole observation period. Ta was relative stable, with a mean value of 15.4°C, compared to Rn. However, Ta in April was relatively low, increased gradually later, reached the peak in the beginning of August.

### 2.2 Characteristics of Farmland ET in the Semi-arid Area of the Loess Plateau

**Figure 2:**
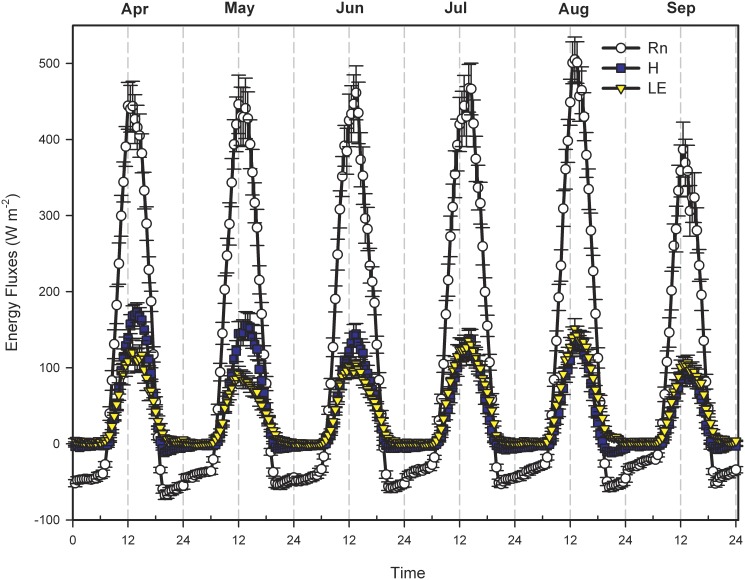
Monthly averaged diurnal variations of energy fluxes over the semi-arid farmland ecosystem in the Loess Plateau. Error bars represent one standard error. Rn, net radiation (W m^−2^); H, sensible heat flux (W m^−2^); LE, latent heat flux (W m^−2^).

According to Figure 2, the diurnal variation of monthly latent heat flux during the study period was character as “single-peak” curve, without the “ET highland” phenomenon around noon that reported by Guo *et al*. [28] in the winter wheat farmland. For the nighttime, latent heat flux was lower with relative stable, but it increased gradually after sunrises, reaching the peak around noon, which then decreased again and became stable till sunset. The latent heat flux peaks appeared between 12∶00 and 14∶00. The latent heat flux peaks appeared about 12∶00 in April and May, while it appeared about one and half hour later (about 13∶30) in June, July, August and September. Besides, there were significant differences between daily peaks of latent heat flux in each month, with the highest daily peak appeared in August while the lowest daily peak appeared in May. The latent heat flux peak values from April to September were 121.2, 92.3, 105.2, 135.2, 151.4 and 106.9 W m^−2^ respectively. The peak value of latent heat flux and H lagged about one hour later that of Rn. The daily variations of three components (Rn, latent heat flux and sensible heat flux) of energy balance didn’t lie in same phase, which may be related with their different ways of energy transmission. The main energy resources of Rn (solar radiation, atmospheric radiation and earth surface radiation) were transmitted through electromagnetic wave, while that of latent heat flux and sensible heat flux was mainly transmitted through atmospheric turbulence with the later transmission far slower than the former one. Moreover, the different physical measure plane of radiation sensor and EC system may be another important reason causing the phase differences of energy components [29].

**Figure 3:**
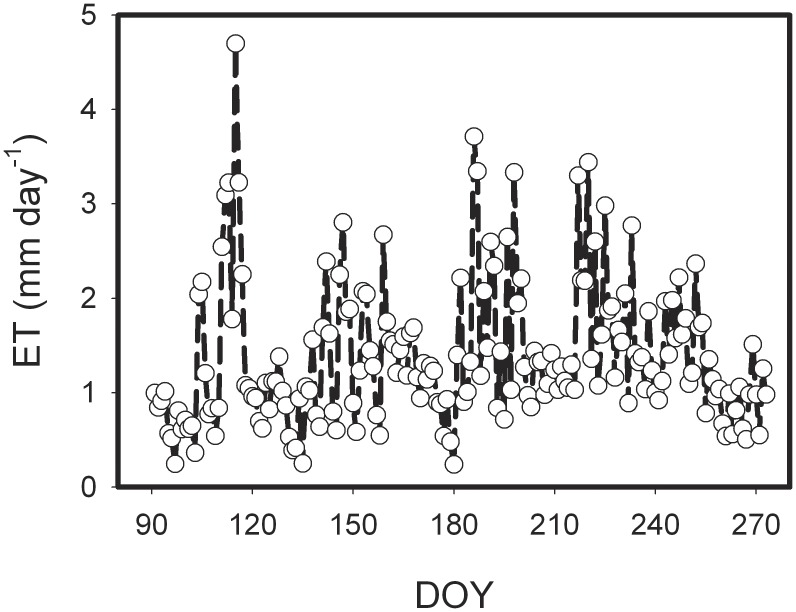
Seasonal variations of evapotranspiration (ET) over the semi-arid farmland ecosystem in the Loess Plateau.

There was significant seasonal variation in daily ET rate of the semi-arid farmland ecosystem in the Loess Plateau (Figure 3), with the maximum daily ET rate of 4.69 mm day^−1^ (DOY 115), minimum daily ET rate of 0.24 mm day^−1^ (DOY 180) and the mean daily ET rate of 1.38±0.75 mm day^−1^ during the whole growing season. Furthermore, there was obvious difference in monthly ET, with relatively high in July and August, but low in May and September. The accumulative ET during the study period was 252.4 mm, lower than 11.7% precipitation and significantly lower than the averaged precipitation over years in the same period (329.3 mm, 1979 to 2008 observation data from Dingxi weather station), which indicated that the farmland ET mainly derived from natural rainfall during 2010 growing season in the semi-arid area of the Loess Plateau.

**Figure 4:**
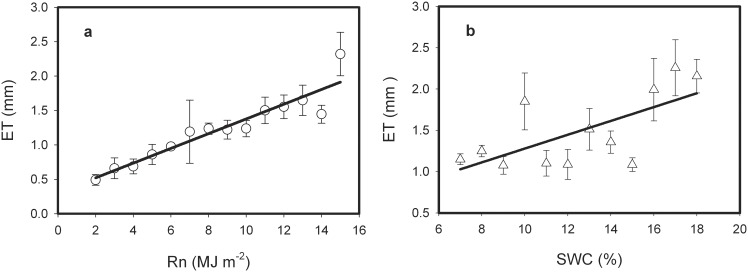
The response of evapotranspiration (ET) to net radiation (Rn) and soil water content (SWC) over the semi-arid farmland ecosystem in the Loess Plateau. ET data were averaged with Rn bins of 1^−2^ (a), and SWC bins of 1% (b), respectively, during the growing season over the semi-arid farmland ecosystem in the Loess Plateau. Error bars represent one standard error.

Seasonal variations in abiotic variables (Rn, SWC, Ta, and VPD) and biotic variables (LAI) exert regulations on water exchange between atmosphere and terrestrial ecosystems [30], [31]. Rn was the main environmental factor of ET in the semi-arid farmland of the Loess Plateau, followed by SWC (Figure 1, 3). ET was highly linked with net radiation (Rn), and there was a significant linear relationship between ET and Rn. Rn explained 88% of the variances in daily ET (regression analysis, p<0.001, Figure 4a). Similar environmental controls were found by Yu *et al*. [32] with Rn as the main driver of ET in a wetland and seasonal patterns of ET closely following radiation. Similarly, a linear increase of ET with SWC was observed (Figure 4b). Nevertheless, SWC explained only 44% of the variance in ET.

### 2.3 Characteristics of Farmland Crop Coefficient in the Semi-arid Area of the Loess Plateau

**Figure 5:**
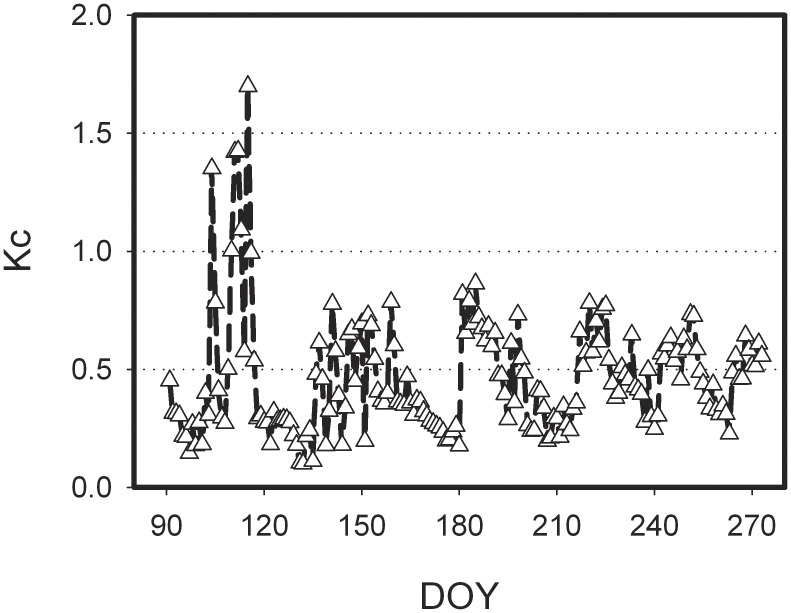
Seasonal variation of crop coefficient (Kc) over the semi-arid farmland ecosystem in the Loess Plateau.

The farmland Kc values during the growing season in the semi-arid area of the Loess Plateau showed the obvious seasonal fluctuation (Figure 5), with relatively higher in the middle and late April. There were six days with over 1.0 of Kc value, and the Kc value on 25^th^ April even reached 1.70. However, Kc decreased dramatically in the beginning of May, which valued less than 1 and mainly fluctuated near 0.50 in the following months (from June to September). Therefore, rainfall events will exert obvious impact on the seasonal variation of Kc. The Kc reached the lowest value (0.10) in 12^th^ May due to the less rainfall and drought weather in the middle of May. The averaged Kc during the whole observation period was 0.46±0.25 (mean ± standard deviation).

### 2.4 Relationship between Kc and Environmental Factors

The Pearson correlation analysis between Kc and major environment factors showed that Ws was the most important in controlling Kc of semi-arid farmland (p<0.01). RH and VPD also showed very significant correlation to Kc (p<0.01), so as SWC (p<0.05) at the 95% confidence level. However, Kc were no significant correlation with Rn and Ta (p>0.05) (Table S1). Based on the correlation analysis results, Kc can be expressed by its four significant correlated environmental factors statistically, Ws, RH, SWC and VPD (Eq. 3):
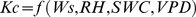
(3)


**Figure 6:**
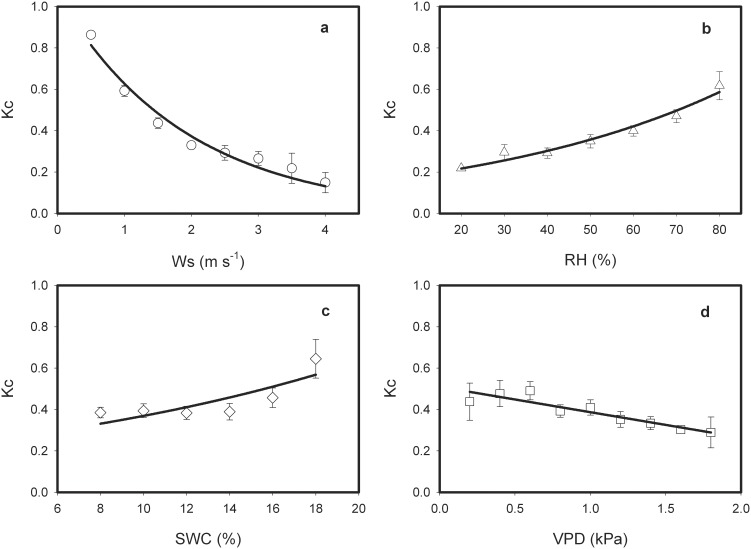
The response of crop coefficient (Kc) to wind speed (Ws), relative humidity (RH), soil water content (VPD), and vapor pressure deficit (VPD), respectively. Kc data were averaged with Ws bins of 0.5^−1^ (a), RH bins of 10% (b), SWC bins of 2% (c) and VPD bins of 0.2 kPa, respectively, during the growing season over the semi-arid farmland ecosystem in the Loess Plateau. Error bars represent one standard error.

The response ways of Kc to Ws, RH, SWC and VPD were shown in Figure 6. For the sake of analysis expediently, Ws, RH, SWC and VPD were grouped into bins with the following criterion: 0.5 m s^−1^, 10%, 2%, and 0.2 kPa, respectively. According to the regression analysis result, Kc decreased exponentially with Ws, but increased exponentially with RH and SWC, and decreased linearly with VPD (Figure 6, Table 1).

**Table 1:** Regression analysis between daily crop coefficient (Kc) and mainly environmental factors over the semi-arid farmland ecosystem in the Loess Plateau.

Factors	Regression equation	n	R^2^	F	P
Ws (m s^−1^)	Kc = 1.06exp(–0.52Ws)	8	0.97	182.0	<0.0001
RH (%)	Kc = 0.16exp(0.02RH)	7	0.86	132.9	<0.0001
SWC (%)	Kc = –0.22exp(0.05SWC)	6	0.67	8.0	<0.05
VPD (kPa)	Kc = 0.51–0.12VPD	9	0.85	38.6	<0.001

Ws, wind speed (m s^−1^); RH: air relative humidity (%); SWC, soil water content (%); VPD, vapor pressure deficit (kPa).

### 2.5 Development of Kc Model

Based on the correlation and regression analysis between Kc and its major environmental factors, an empirical daily Kc model for the semi-arid farmland in the Loess Plateau that driven by Ws, RH, SWC and VPD, could be given by the following equation:
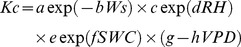
(4)


**Figure 7:**
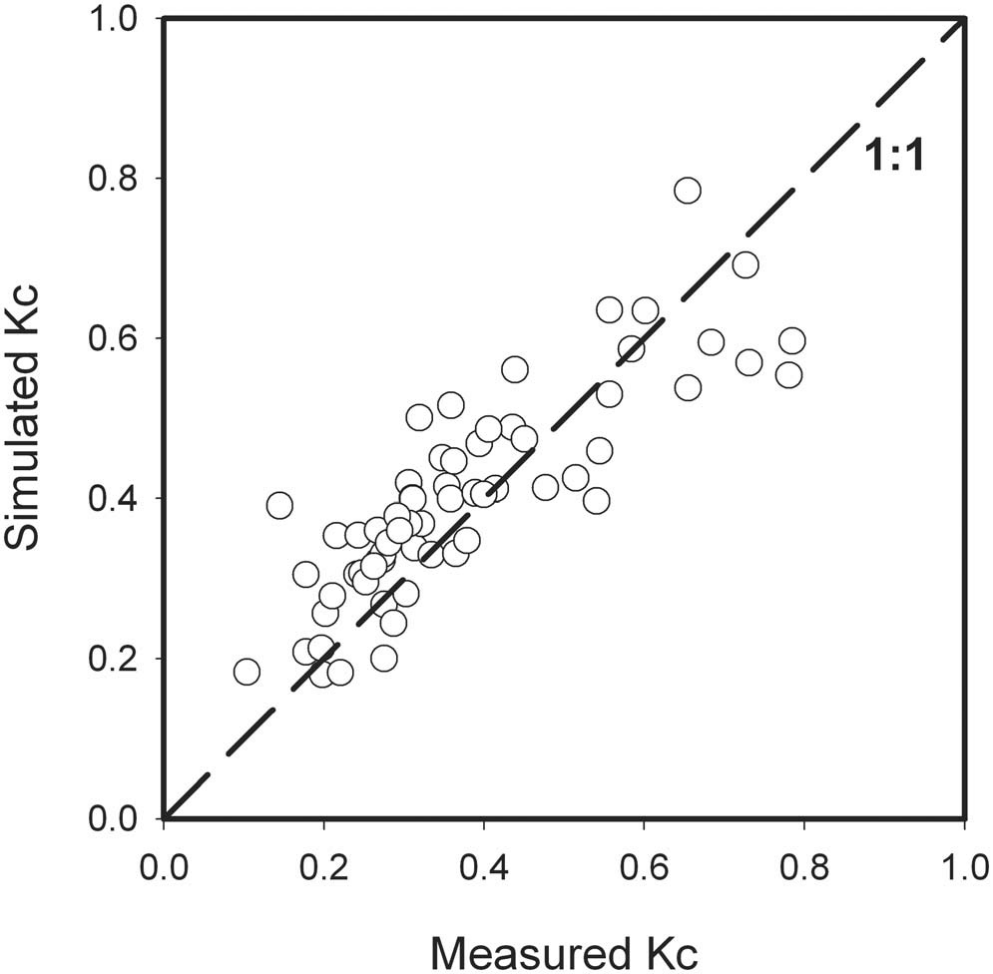
Comparison of simulated and measured crop coefficient (Kc) values.

After mathematical deduction, and then:
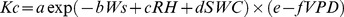
(5)where a, b, c, d, e, and f are fitting parameters, Ws is wind speed; RH is relative air humidity; SWC is soil water content; VPD is vapor pressure deficit; and Kc is crop coefficient. The data of clear days (n = 132) during 2010 growing season was divided into two groups according to the order of Kc magnitude alternately, one data group for fitness, and another data group for verification. Based on daily environmental data and Kc data for the growing season in 2010, an empirical Kc model was developed (n = 66, R^2^ = 0.70):



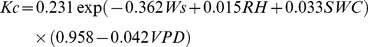
(6)Eq. 6 can be considered as the empirical model for farmland Kc estimating in the semi-arid area of the Loess Plateau. Then, another group of data during the growing season 2010 was used to validate the empirical Kc model (Eq. 6). Statistical indices of root-mean-square error, index of agreement, coefficient of determination and regression coefficient through the origin were 0.09, 0.90, 0.67, and 1.00, respectively, indicating that this Kc model can be able to well describe the Kc dynamics (Figure 7). However, it can be seen from Figure 7 that some scatters discussive from the 1∶1 line, which might be that the biotic factors were not took into account in the Kc model, such as LAI, biomass.

### 2.6 Magnitude of ET and Kc with other Agroecosystems

The peak spring wheat ET value (4.69 mm day^−1^) of the semi-arid farmland ecosystem in the Loess Plateau was approached to the peak ET value of 4.47 mm day^−1^ for the winter wheat on a loess loam soil in a semi-humid region of the northwest China reported by Kang *et al.* [33]. However, the peak ET in the Loess Plateau farmland ecosystem was lower than that of the irrigated winter wheat in the semi-humid region of the North China Plain, which between 6.6 and 7.8 mm day^−1^ found by Lei *et al.* [34], and as large as 6–9 mm day^−1^ reported by Liu *et al.* [35]. The peak spring wheat ET value was also lower than that of 7.0 mm day^−1^ for the cultivated wheat ecosystem in north-central Oklahoma USA [36]. In the semi-arid area of the Loess Plateau, the drought weather condition and few rainfalls, constrained the soil water availability for plant growth, may be the main environmental limiting factor for the farmland ecosystem ET. Moreover, the lack of irrigation management in the traditional farming system in the study region could also result in the relative low ET.

Li *et al.* [37] reported that the spring wheat Kc in an Inner Mongolia of China was 0.55 (initial stage), 1.03 (crop development stage), 1.19 (mid-season stage) and 0.65 (late-season stage), respectively, indicating the average Kc higher than that of 0.46 during the whole season in the Loess Plateau. The average Kc from this study was also significantly lower than 0.93 for winter wheat and 1.1 for corn in North China Plain found by Liu *et al*. [35]. The relatively low Kc value may be related with the lower precipitation and ET in the semi-arid area of the Loess Plateau. However, the averaged Kc was higher than Kc value reported for grassland field in the Liudaogou basin of the Loess Plateau (below 0.3) [38]. Based on the obvious seasonal variation of daily Kc value, it is necessary to be cautious about applying Kc as a constant for ET estimation in the Loess Plateau. The Kc variability might explain possibly that the lower ET estimation derived from Penman-Monteith model with fixed Kc values than that observed through eddy covariance and lysimeter in the Loess Plateau reported by Zhang *et al*. [18].

## Conclusions

The diurnal latent heat flux of the semi-arid farmland ecosystem in the Loess Plateau showed a single-peak shape. There was significant variance in daily ET rate with a maximum daily ET rate of 4.69 mm day^−1^. The relative low daily ET could be related to the drought weather condition and lack of irrigation in study region. Radiation was the main driver of ET of the semi-arid farmland ecosystem in the Loess Plateau. Cumulative ET during the study period estimated directly by eddy covariance method was lower than precipitation received during the same period in this farmland ecosystem. The farmland Kc values showed the seasonal fluctuation, and significantly correlated with Ws, RH, SWC and VPD. Regression analysis results showed that Kc decreased exponentially with increasing Ws, increased exponentially with RH and SWC, and decreased linearly with increasing VPD. The four environmental factors can explain most of the day-to-day variation in Kc, and then an empirical daily Kc model for the semi-arid farmland ecosystem in the Loess Plateau was developed, which showed a good performance in consistency between the simulated and measured Kc.

## Supporting Information

Table S1 Pearson product-moment correlation coefficients between daily crop coefficient (Kc) and daily average values for other variables: on a daily basis for clear days during growing season of 2010 over the semi-arid farmland ecosystem in the Loess Plateau.
(DOC)
