# Supplementary material for: Evapotranspiration Measurement and Crop Coefficient Estimation over a Spring Wheat Farmland Ecosystem in the Loess Plateau
Source: PLoS One. 2014 Jun 18;9(6):e100031. doi: 10.1371/journal.pone.0100031 (PMC4062470; doi:10.1371/journal.pone.0100031)
Supplement: Table S1 — Pearson product-moment correlation coefficients between daily crop coefficient (Kc) and daily average values for other variables: on a daily basis for clear days during growing season of 2010 over the semi-arid farmland ecosystem in the Loess Plateau. (DOC) [file pone.0100031.s001.doc]

Supporting Information

Table S1 Pearson product-moment correlation coefficients between daily crop coefficient (Kc) and daily average values for other variables: on a daily basis for clear days during growing season of 2010 over the semi-arid farmland ecosystem in the Loess Plateau.

|  | Kc | Rn | Ta | Ws | VPD | SWC | RH |
| --- | --- | --- | --- | --- | --- | --- | --- |
| Kc | 1.000 |  |  |  |  |  |  |
| Rn | -0.012 | 1.000 |  |  |  |  |  |
| Ta | -0.117 | 0.304^**^ | 1.000 |  |  |  |  |
| Ws | -0.681^**^ | 0.070 | -0.018 | 1.000 |  |  |  |
| VPD | -0.356^**^ | 0.497^**^ | 0.628^**^ | -0.034 | 1.000 |  |  |
| SWC | 0.263^*^ | 0.015 | -0.327^**^ | -0.018 | -0.142 | 1.000 |  |
| RH | 0.439^**^ | 0.309^**^ | -0.035 | -0.088 | -0.739^**^ | -0.143 | 1.000 |

Rn, net radiation; Ta, air temperature; Ws, wind speed; VPD, atmospheric vapor pressure deficit; SWC, soil water content; RH, air relative humidity.
